# Suicidal ideation and associated factors among high school adolescents

**DOI:** 10.11606/s1518-8787.2020054001637

**Published:** 2020-03-25

**Authors:** Cyntia Meneses de Sá Sousa, Márcio Dênis Medeiros Mascarenhas, Keila Rejane Oliveira Gomes, Malvina Thaís Pacheco Rodrigues, Cássio Eduardo Soares Miranda, Karoline de Macêdo Gonçalves Frota

**Affiliations:** I Universidade Federal do Piauí Programa de Pós-Graduação em Saúde e Comunidade TeresinaPI Brasil Universidade Federal do Piauí, Programa de Pós-Graduação em Saúde e Comunidade, Teresina, PI, Brasil

**Keywords:** Adolescent, Suicidal Ideation, Risk Factors, Sex Offenses, Child Abuse, Sexual, Cross-Sectional Studies

## Abstract

**OBJECTIVE:**

To analyze the prevalence of suicidal ideation and its associated factors in school adolescents.

**METHODS:**

Cross-sectional school-based study with 674 students from public and private schools in Teresina, Piauí, in 2016. Bivariate analysis was performed with the chi-square test and multiple analysis by the Poisson regression model to estimate prevalence ratios (PR) and 95% confidence intervals (95%CI).

**RESULTS:**

The study participants were mostly female (56.7%), black (77.4%), who lived with their parents (85%), whose mothers had schooling greater than or equal to 8 years of schooling (68.8%), with family income greater than a minimum wage (58.3%), practitioners of some religion (86.8%) and coming from public school (64.7%). The prevalence of suicidal ideation was 7.9%. Higher frequency of suicidal ideation was reported among female students (10.2%). Suicidal ideation was statistically associated with students who reported not living with their parents (adjusted PR = 2.27; 95%CI 1.26–4.10; p < 0.05) and those who reported having suffered sexual violence by other students, teachers or school staff (adjusted PR = 3.40; 95%CI 1.80–6.44; p < 0.05), among which the prevalence of suicidal ideation was more than three times that observed among those who did not mention this type of violence.

**CONCLUSION:**

The prevalence of suicidal ideation in school adolescents was associated with female students, who did not live with parents and have been victim of sexual violence at school. We recommend advising the school community and health professionals to identify signs of suicidal behavior, especially in those with suspicion or proof of the occurrence of sexual violence at school.

## INTRODUCTION

Adolescence is a period of complex development, during which individuals can assume different risk habits, including suicidal behavior, which covers suicidal ideation, suicide attempts and suicide itself^1,2^. Among adolescents, the factors that favor suicidal behavior pointed out in the literature are: poverty, violence, economic differences, family turmoil, use of psychoactive substances, little social support, love disappointment, homosexuality, loneliness, family history of suicidal behavior, prior attempt and suicidal ideation^3-6^.

Suicide is a global public health problem, with an increase in the number of attempts and deaths. Currently, it is the second cause of death in the population aged between 15 and 29 worldwide^7^. In Brazil, between 2002 and 2012, suicide deaths increased by 33.6% in the general population and 15.3% in the group of young people^8^.

Teresina, capital city of the state of Piauí, showed a 41.6% increase in the number of suicides among the general population, from 41 deaths in 2006 to 58 deaths in 2015^9^. In the last year, the overall suicide mortality rate in Teresina (6.9/100,000 inhabitants) was higher than the national rate (5.5/100,000 inhabitants)^9^. Regarding suicide deaths among adolescents aged between 10 and 19 years, Teresina presented a mortality rate of 2.1/100,000 inhabitants in 2015, being the 17th when compared with other Brazilian capitals that year^9^.

In addition to the suicidal phenomenon itself, the ideation of the act is a major challenge and threat to the health of adolescents^3,4^. A study conducted in 32 countries in the Americas with students aged between 13 and 17 showed a prevalence of suicidal ideation of 16.2% among women and of 12.2%^10^among men. Regarding the prevalence of suicidal ideation, studies conducted with adolescents in Brazil^11^and Canada^12^ found a prevalence of 7.7% and 10.8% respectively, showing that the problem is part of the national and foreign reality.

Although still little explored in the literature, sexual abuse has a strong association with suicidal ideation among adolescents^13,14^. Depending on situations in which sexual violence occurs (age of the victim, perpetrator, time of abuse, place of occurrence and affective bond), the consequences can generate psychological disorders, considered important predictors for the development of suicidal ideation^13,15^. A cross-sectional population-based study conducted with young people aged between 18 and 24 in the municipality of Pelotas (RS) showed that young victims of sexual abuse had a higher risk of suicide when compared with those who did not suffer such violence^15^.

Thus, given the growth of suicide mortality rates worldwide and the scarce information about suicidal ideation among high school adolescents in the municipality of Teresina, knowing the frequency of this problem and the factors associated with young students is necessary to recommend appropriate preventive measure. In this context, our study sought to analyze the prevalence of suicidal ideation and associated factors in high school adolescents in the municipality of Teresina (PI).

## METHODS

This is a cross-sectional school-based study, with students aged between 14 and 19 years old, regularly enrolled in high school from public and private schools in the urban area of the municipality of Teresina, Piauí. Our study is part of a study entitled “Health in school: situational diagnosis in high school.”^16^ The study was developed by teachers and students of a graduate program in the area of collective health. Details of the methodology used in the base study are available in the literature^16^.

**Figure 1:**
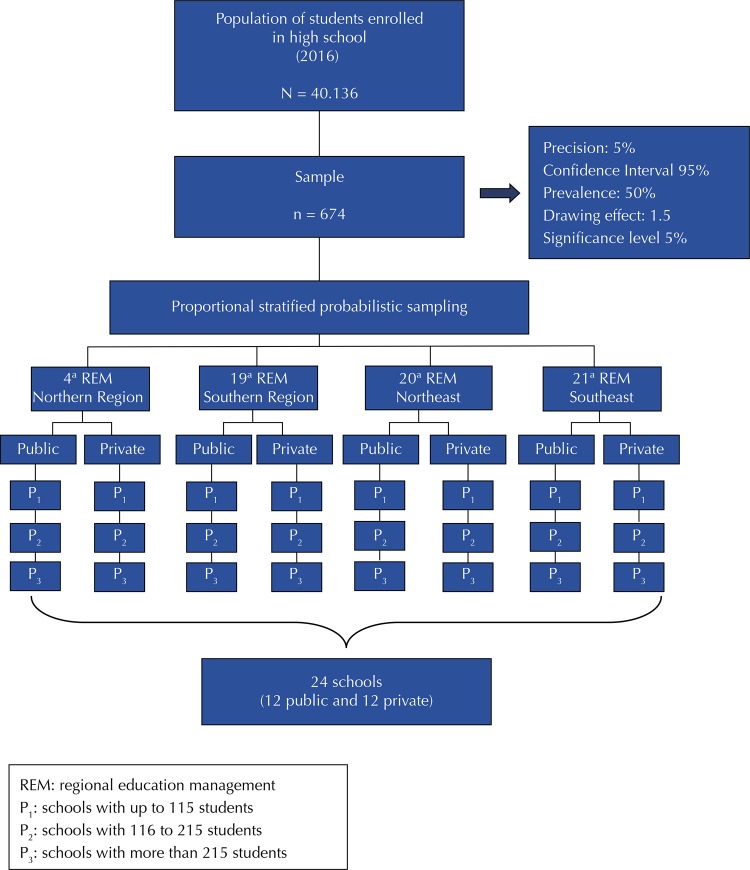
Flowchart of the distribution of the sample of high school students in public and private schools of Teresina, Piauí, 2016.

We used the proportional stratified probabilistic sampling^17^, estimated in the Epi Info 6.04d program (Centers for Disease Control and Prevention, Atlanta, United States) to select the students, considering the population of high school students from private and public schools (N = 40,136), according to data from the 2014 School Census^18^. A 95% confidence interval (95%CI) was adopted, a 50% prevalence was adopted for outcomes of interest for the survey, as well as a 5% accuracy, an 1.5 drawing effect and a 5% significance level^19^. Thus, the minimum sample was estimated in 571 adolescents, with 20% (114) added to the possibility of losses and refusals, totaling a final sample of 685 adolescents, as shown in Figure 1. Despite the refusal of 11 students (1.6%) in participating in our study, the sample size was not compromised, with no sample loss.

The selection of schools considered the type of administration (public and private), geographical location (teaching managers – South, Southeast, Northeast and North) and the size (small: up to 115 students; average: 116–215 students; high: more than 215 students). A public and a private school of each size were selected, distributed in each of the city’s four regional teaching managers, totaling 12 public schools and 12 private schools, also shown in Figure 1. The sample was distributed in schools chosen proportionally to the number of students existing according to the school size, high school grade, sex and age.

Data were collected in between June and November of 2016. The time of data collection was determined by the direction of each school, according to the adequacy to the calendar and hours of classes of the institution. The questionnaire for data collection was self-applicable and composed of six blocks of questions: 1. sociodemographic aspects; 2. sexual initiation; 3. objective and perceived knowledge; 4. vaccine aspects; 5. Nutritional aspects and 6. violence and insecurity at school. Questions from blocks 1 and 6 were used in our study, and the questions contained in block 6 were adapted from the questionnaire used in the *Pesquisa Nacional de Saúde do Escolar* (PeNSE – National School Health Survey) of 2012^20^ and the Victimization Survey used by Lecoque^21^, whose purpose was to verify the phenomena of violence existing in the school.

Those who answered “yes” to at least one of the following questions were considered as victims of sexual violence: a) “Within the last 12 months, have you felt sexually harassed by other students at school?”; b) “Within the last 12 months, have you been sexually harassed by teachers or staff at school?”; c) “Within the last 12 months, have you been forced or seduced to engage in sexual acts (or have been the victim of sexual violence) by other students at school?”; d) “Within the last 12 months, have you been forced or seduced to engage in sexual violence by teachers or staff at school?”.

The outcome of the study (prevalence of suicidal ideation) was obtained through the answer “yes” to the following question: “Within the last 12 months, have you seriously thought about committing suicide (taking your own life)?”.

Data were subjected to double typing in the Epi Info program to check for possible inconsistencies and, when necessary, perform the appropriate corrections. Statistical analysis was performed using the software SPSS 20.0 (Statistical Package for the Social Sciences).

Descriptive analysis was performed with presentation of the distribution of relative and absolute frequencies. The inferential analysis was performed to determine the association between the prevalence of suicidal ideation and independent variables by Pearson chi-square and Fischer’s exact tests (when applicable), considering a 5% level of significance. The Mantel-Haenszel method was used for cross-sectional studies to estimate the crude prevalence ratio (crude PR). The Poisson regression model with robust variance and 95%CI was used to estimate the adjusted prevalence ratio (adjusted PR), in which all independent variables were included, considering the level of significance of p < 0.05. The power of the test was estimated in OpenEpi version 3.01.

Our study was approved by the Research Ethics Committee of UFPI (opinion no. 1,495,975). The Secretary of State for Education and Culture of Piauí and the managers of private schools authorized the study in schools under their responsibility. All participants of the study signed the Informed Consent Form and the Informed Assent Form when necessary.

## RESULTS

The study included 674 adolescents (refusal of 11 students, 1.6%), whose mean age was 16.4 years (SD = 1.2). The sample consisted predominantly of female students (56.7%), black (77.4%) and students who lived with their parents (85.0%). Regarding the mother’s schooling, 68.8% of them had more than eight years of schooling. Most students said they did not perform paid activity (83.1%), having family income greater than a minimum wage (58.3%) and practice a religion (86.8%). Almost seven out of ten students were from public schools (64.7%) and most of then attended the 2nd year of high school (35.9%), as shown in Table 1.

**Table 1:** Characterization of high school students from public and private schools in Teresina, Piauí, 2016.

Variables	N	%
Total	674	100.0
Age (in years)		
14 to 16	346	51.3
17 to 19	328	48.7
Sex		
Female	382	56.7
Male	292	43.3
Skin color		
Black^a^	522	77.4
Other^b^	152	22.5
Live with parents?		
Yes	573	85.0
No	101	15.0
Mother’s schooling (years of study)		
< 8 years	210	31.2
≥ 8 years	464	68.8
Paid activity?		
Yes	114	16.9
No	560	83.1
Household income		
Up to 1 minimum wage	281	41.7
More than 1 minimum wage	393	58.3
Religion		
Yes	585	86.8
No	89	13.2
Type of school		
Public	436	64.7
Private	238	35.3
School Year		
HS Freshman^c^	222	32.9
HS Sophomore	242	35.9
HS Junior	210	31.2

HS: High School

^a^ Includes black and brown

^b^ Includes white, yellow and indigenous people

The prevalence of suicidal ideation within the last twelve months prior to the study was 7.9%, with a higher frequency in female students (10.2%). In the crude analysis, suicidal ideation was associated with female students, twice as often as observed in males (crude PR = 2.13; 95%CI 1.18–3.85; p < 0.05; power = 66.85%), reaching the significance threshold in the adjusted analysis (adjusted PR = 1.87; 95%CI 0.96–3.62; p = 0.052), as shown in Table 2.

**Table 2:** Prevalence of suicidal ideation according to sociodemographic and historical aspects of violence within the last 12 months before the study among high school students from the public and private network of Teresina, Piauí, 2016.

Variable	Total	Suicidal ideation		Gross PR		Adjusted PR^a^	
			
	(n = 674)	n	%	95%CI	p-value^b^	95%CI	p-value^c^
Suicidal ideation	674	53	7.9	-	-	-	-
Age (in years)					0.097		0.402
14 to 16	346	33	9.5	1.56 (0.92–2.67)		1.31 (0.70–2.45)	
17 to 19	328	20	6.1	1		1	
Sex					< 0.05		0.052
Female	382	39	10.2	2.13 (1.18–3.85)		1.87 (0.96–3.62)	
Male	292	14	4.8	1		1	
Skin color					0.290		0.330
Black^d^	522	38	7.3	1		1	
Other^e^	152	15	9.9	0.74 (0.42–1.30)		0.76 (0.43–1.33)	
Live with parents?					< 0.05		< 0.05
No	101	15	14.9	2.24 (1.28–3.92)		2.27 (1.26–4.10)	
Yes	573	38	6.6	1		1	
Mother’s schooling (years of study)					0.278		0.191
≥ 8 years	464	40	8.6	1.39 (0.76–2.55)		1.55 (0.81–2.97)	
< 8 years	210	13	6.2	1		1	
Paid activity?					0.453		0.506
No	560	46	8.2	1.34 (0.62–2.89)		1.28 (0.62–2.66)	
Yes	114	7	6.1	1		1	
Household income					0.793		0.732
Up to 1 minimum wage	281	23	8.2	1.07 (0.64–1.81)		1.10 (0.63–1.91)	
More than 1 minimum wage	393	30	7.6	1		1	
Religion					0.205		
No	89	10	11.2	1.53 (0.80–2.93)		1.27 (0.64–2.51)	0.495
Yes	585	43	7.4	1		1	
Type of school					0.266		0.279
Public	436	38	8.7	1.38 (0.77–2.46)		1.38 (0.77–2.50)	
Private	238	15	6.3	1		1	
School Year							
HS Freshman	222	25	11.3	0.71 (0.36–1.39)	0.313	1.86 (0.86–4.03)	0.117
HS Sophomore	242	17	7.0	1.74 (0.83–3.62)	0.136	1.17 (0.53–2.58)	0.701
HS Junior	210	11	5.2	1		1	
Victim of sexual violence					< 0.05^f^		< 0.05
Yes	43	12	27.9	4.30 (2.44–7.55)		3.40 (1.80–6.44)	
No	631	41	6.5	1		1	
Victim of physical violence					0.123^f^		0.22
Yes	25	4	16.0	2.12 (0.83–5.41)		1.96 (0.67–5.74)	
No	649	49	7.6	1		1	

PR: prevalence ratio (Mantel-Haenszel method); 95%CI: 95% confidence interval; HS: high school

^a^ adjusted PR for all independent variables (Poisson regression).

^c^ chi-square test (95%CI).

^b^ Poisson regression with robust variance (95%CI).

^d^ Includes black and brown

^e^ Includes white, yellow and indigenous people.

^f^ Fisher’s exact test (95%CI).

The presence of suicidal ideation was statistically associated with students who reported not living with parents (adjusted PR = 2.27; 95%CI 1.26–4.10; p < 0.05; power = 77.23%) and those who reported having suffered sexual violence by other students, teachers or school staff (adjusted PR = 3.40; 95%CI 1.80–6.44; p < 0.05; power = 99.82%). The frequency of reference to suicidal ideation among students who suffered some type of sexual violence within the school was more than three times that of students who did not suffer this type of violence, as shown in Table 2.

Although no significant association was found, it is important to emphasize that the higher prevalence of suicidal ideation were observed among younger students (9.5%), with mother with a high education level (8.6%), without paid activity (8.2%), with low family income (8.2%), no religion (11.2%) and from public school (8.7%). Moreover, although without statistical significance, thoughts about suicide were reported among high school students who suffered physical violence at school (16%) when compared with those who were not victims of this violence (7.6%), also shown in Table 2.

## DISCUSSION

Our study shows results of the prevalence of suicidal ideation among school adolescents, enabling the identification of associated factors. We observed a 8% of suicidal ideation rate within the 12 months before our study, which shows an association with some sociodemographic factors and sexual violence suffered at school. A similar prevalence was found in a population-based study conducted with 960 adolescents in the city of Pelotas (RS)^11^ and also in Otawa (Canada)^12^. Both studies show characteristics similar to this study, targeting adolescent students and outcome measured within the 12 months before the study, which makes this study consistent with the world reality.

Among the sociodemographic characteristics, the female sex and the fact that they did not live with the parents presented a statistically significant association with suicidal ideation. Although the association with the female variable is not seen in the adjusted analysis, reaching the limit of statistical significance, the prevalence of suicidal ideation was higher in female students, which corroborates evidence already pointed out in other studies that girls are more prone to suicidal ideation^3,10,12^. Such studies have shown that, although boys succeed in their suicide attempts in a greater proportion, girls have a higher frequency of thoughts about the act^3,10,12^.

One of the factors related to the higher prevalence of suicidal ideation among girls may be linked to the fact that girls enter earlier in the puberty phase, experiencing the physical changes and pressures proper to the period before the boys, especially changes related to what is expected of their behavior in society and the family repressions girls suffer^3,10,12^. The feeling of loneliness and concern about family problems perceived in the daily lives of girls were reported in a study conducted by Reis et al.^22^ as a situation of worsening of mental problems. Moreover, girls present more recurrent psychological problems, such as depression, mood disorder, anxiety and introspection, factors that are strongly related to the onset of suicidal ideation^3,23^.

In this investigation, suicidal ideation was more prevalent among adolescents who did not live with their parents. The absence of affection and family support reveal a family context often without communication, which can generate feelings of abandonment and insecurity^24^. These feelings become the basis for the beginning of depression, which in turn is a strong factor for the emergence of suicidal behaviors, including ideation^23,24^. Parental care is the main basis for the good social and mental development of adolescents^23,24^. Therefore, the parental company is an emotional bond that must be developed with quality since childhood to ensure the development of adolescents with psychological maturity and able to face the different emotional and/or mental instabilities with more confidence^25^.

Another factor that stood out in our study was the greatest report of suicidal ideation among victims of sexual violence at school. This finding was also reported in a study conducted in Mexico in 2010^14^, in which the history of sexual abuse at school increased the likelihood of suicidal ideation by 92%. In Brazil, a study conducted in the municipality of Pelotas showed that the risk of suicide throughout life was present in 29.2% among individuals who suffered sexual violence participating in the survey^15^.

Sexual violence, in the form of sexual exploitation or abuse, causes mental and physical consequences to the victim, regardless of their age group^13^. It can be practiced by people who have affective (intrafamily) ties with the victim or by people who are not related or do not coexisted with the victim (extrafamily)^13^.

Sexual violence is evidently more frequent among female victims^15,26,27^. Epidemiological analysis on sexual violence conducted in Brazil showed that 92.4% of sexual violence suffered by adolescents from 2011 to 2017 and reported in the *Sistema de Informação de Agravos de Notificação* (SINAN – Notification Diseases Information System) occurred with women, with the residence being the most frequent place (58.7%), followed by the public route (14.1%) and school (1.2%)^28^. The higher occurrence of sexual violence in women may be related to a historical and sociocultural context in which women were raised and educated to believe they should be submissive and accept any kind of domination^29^.

Any type of violence impacts the lives of the people who experience it and affects integral development in adolescents, impairing family and social well-being^29^. Regarding the consequences of sexual violence, they can be physical and psychological and manifest both in the short-term (sleep and feeding disorders, excessively frequent baths, repeated gestures, anxious conditions, isolation, shame, fear and depression, among others) and in the long term (alcohol and other drug abuse, promiscuity, sexual dysfunctions, low self-esteem, menstrual dysfunctions and suicidal behaviors)^13,25^. Depression, one of the factors that generate suicidal behavior among adolescents, is one of the most recurrent manifestations among victims of sexual violence and may have repercussions at any stage of life, causing situations of emotional, social and family stress^15,26^.

The school is considered a learning space, providing better social equality and exerting a strong influence on the formation of students^30^. The experiences of daily school will serve as the basis for the process of human formation of children and adolescents, who should fully participate in these activities, in order to achieve a complete development^30^. Thus, the school should be understood by the students as a safe and welcoming place, serving as a means for social, cultural and cognitive development, and it should not therefore be where students face any type of violence, which can generate stressful situations that result in suicidal ideations, one of the most severe and traumatic consequences for an individual^3,27,30^.

Suffering any kind of violence, including sexual, in the school environment, in addition to the consequences listed earlier, results in losses in learning, because a student who is victim of violence, whether by teachers or other students, will feel fear or shame to return to school, thus hindering learning and discouraging the intention of continuing the studies^26,30^. In addition to educational impairments, being a victim of sexual violence at school can cause social damage, because the delay in studies can generate feelings of low self-esteem, exclusion and isolation, as well as damage in the family environment, since family members experience pain and shame along with the victim^26,27,30^.

Our findings evidence the importance of investigating suicidal ideation among school adolescents, considering that the outcome is related to several other problems and can generate greater consequences. We approached a current and growing problem, which has serious consequences for the whole society. A strong association and higher frequency of suicidal ideation among female adolescents and among those who did not live with their parents was shown, and the high reference to suicidal thinking among students who reported sexual violence within the school stood out in our study.

We cite some facts that could bias our study: a) information bias, because the subject “suicidal ideation” is surrounded by taboos and prejudices, which may have led some participants to answer incomplete or even fail to answer some questions, affecting the estimate of the prevalence of outcomes studied; b) memory bias, since it is not possible to ensure that all respondents remembered exactly all aspects investigated. Moreover, suicidal ideation is very subjective and influenced by multiple factors that have not been analyzed in our study, thus requiring a more detailed study. However, these factors did not hinder a proper analysis, considering the methodological design performed in order to control and reduce losses, as well as the findings essential for decision-making in school health management.

The information addressed here reveals situations experienced by students and that should attract the attention of parents, family members, school professionals, health sector managers and society in general for the potential risks arising from the suicide attempt and their association with sexual violence in the school environment. Both are complex identification and approach problems present in our society.

We think it is essential to disseminate and debate qualified information on the subject and develop prevention strategies involving the family, students, teachers and other school staff. Possible signs of suicidal behavior and the possibility of sexual violence in school should be identified so that other problems can be avoided. Given the importance of the theme, we recommend the investigation of questions related to suicidal ideation and associated factors in Brazilian health surveys of national scope, in order to fill the existing knowledge gap when compared with the reality of adolescent in school. A broader picture of the issue can increase existing public policies in facing a complex and rapidly growing problem around the world.
