# Supplementary material for: Suicidal ideation and associated factors among high school adolescents
Source: Rev Saude Publica. 2020 Mar 25;54:33. doi: 10.11606/s1518-8787.2020054001637 (PMC7100950; doi:10.11606/s1518-8787.2020054001637)
Supplement: Translation

# Translation: Ideação suicida e fatores associados entre escolares adolescentes

**Authors:** Cyntia Meneses de Sá Sousa, Márcio Dênis Medeiros Mascarenhas, Keila Rejane Oliveira Gomes, Malvina Thaís Pacheco Rodrigues, Cássio Eduardo Soares Miranda, Karoline de Macêdo Gonçalves Frota

**Affiliations:** I Brasil Universidade Federal do Piauí, Programa de Pós-Graduação em Saúde e Comunidade, Teresina, PI, Brasil

**Keywords:** Adolescente, Ideação Suicida, Fatores de Risco, Delitos Sexuais, Abuso Sexual na Infância, Estudos Transversais

## Abstract

**OBJETIVO:**

Analisar a prevalência de ideação suicida e fatores associados em adolescentes escolares.

**MÉTODOS:**

Estudo transversal de base escolar com 674 estudantes de escolas públicas e privadas em Teresina, Piauí, em 2016. Realizou-se análise bivariada com o teste do qui-quadrado e análise múltipla pelo modelo de regressão de Poisson para estimar as razões de prevalência (RP) e intervalos de confiança de 95% (IC95%).

**RESULTADOS:**

Os participantes do estudo foram em sua maioria estudantes do sexo feminino (56,7%), negros (77,4%), que moravam com os pais (85%), cujas mães apresentavam escolaridade maior ou igual a 8 anos de estudo (68,8%), com renda familiar maior que um salário mínimo (58,3%), praticantes de alguma religião (86,8%) e procedentes de escola pública (64,7%). A prevalência de ideação suicida foi de 7,9%. Maior frequência de ideação suicida foi relatada entre estudantes do sexo feminino (10,2%). Ideação suicida foi associada estatisticamente aos alunos que referiram não residir com os pais (RP ajustada = 2,27; IC95% 1,26–4,10; p < 0,05) e àqueles que informaram ter sofrido violência sexual por outros alunos, professores ou funcionários da escola (RP ajustada = 3,40; IC95% 1,80–6,44; p < 0,05), entre os quais a prevalência de ideação suicida foi mais de três vezes a observada entre aqueles que não referiram esse tipo de violência.

**CONCLUSÃO:**

A prevalência de ideação suicida em adolescentes escolares foi associada ao sexo feminino, não residir com os pais e ter sido vítima de violência sexual na escola. Recomenda-se alertar a comunidade escolar e profissionais de saúde para identificarem sinais do comportamento suicida, em especial naqueles com suspeita ou comprovação da ocorrência de violência sexual na escola.

## INTRODUÇÃO

A adolescência é um período de complexo desenvolvimento, durante o qual os indivíduos podem assumir diversos hábitos considerados de risco, dentre eles o comportamento suicida, que abrange a ideação suicida, tentativas de suicídio e o suicídio propriamente dito^1,2^. Entre adolescentes, os fatores que favorecem o comportamento suicida apontados pela literatura são: pobreza, violências, diferenças econômicas, conturbação familiar, uso de substâncias psicoativas, pouco suporte social, decepção amorosa, homossexualidade, solidão, histórico familiar de comportamento suicida, tentativa prévia e ideação suicida^3-6^.

O suicídio constitui-se em um problema de saúde pública mundial, apresentando aumento no número de tentativas e de mortes. Atualmente, é a segunda causa de morte na população de 15 a 29 anos em todo o mundo^7^. No Brasil, nos anos de 2002 a 2012, as mortes por suicídio aumentaram em 33,6% na população geral e 15,3% no grupo de jovens^8^.

Teresina, capital do estado do Piauí, apresentou incremento de 41,6% no número de suicídios em toda a população, passando de 41 óbitos em 2006 para 58 óbitos em 2015^9^. Nesse último ano, a taxa de mortalidade geral por suicídio em Teresina (6,9/100 mil habitantes) foi superior à taxa nacional (5,5/100 mil habitantes)^9^. Em relação às mortes por suicídio de 10 a 19 anos, Teresina apresentou taxa de mortalidade em 2015 de 2,1/100 mil habitantes, estando em 17ª posição ao se comparar as capitais brasileiras nesse ano^9^.

Além do fenômeno suicida em si, a ideação do ato é um grande desafio e ameaça para a saúde dos adolescentes^3,4^. Estudo realizado em 32 países das Américas com estudantes de 13 a 17 anos de idade mostrou que a prevalência de ideação suicida entre mulheres foi de 16,2% e entre os homens foi de 12,2%^10^. Em relação às prevalências de ideação suicida, estudos realizados com adolescentes no Brasil^11^e no Canadá^12^ encontraram prevalência de 7,7% e 10,8% respectivamente, mostrando que o problema faz parte da realidade nacional e estrangeira.

Embora ainda pouco explorado pela literatura, o abuso sexual apresenta forte associação com a ideação suicida entre adolescentes^13,14^. A depender das situações em que a violência sexual ocorre (idade da vítima, perpetrador, tempo de abuso, local de ocorrência e vínculo afetivo), as consequências podem gerar distúrbios psicológicos, considerados importantes preditores para o desenvolvimento de ideação suicida^13,15^. Pesquisa transversal de base populacional realizada com jovens de 18 a 24 anos na cidade de Pelotas (RS) mostrou que jovens vítimas de abuso sexual apresentaram maior risco de suicídio em relação àqueles que não sofreram tal violência^15^.

Assim, diante do crescimento da mortalidade por suicídio em todo o mundo e da lacuna existente sobre informações acerca da ideação suicida entre adolescentes escolares na cidade de Teresina, faz-se necessário conhecer a frequência desse problema e os fatores associados na população de jovens estudantes para que seja possível recomendar ações adequadas de prevenção. Nesse contexto, o objetivo do estudo foi analisar a prevalência de ideação suicida e fatores associados em adolescentes escolares na cidade de Teresina (PI).

## MÉTODOS

Trata-se de um estudo transversal, do tipo inquérito de base escolar, com estudantes de 14 a 19 anos, regularmente matriculados no ensino médio de escolas públicas e privadas da zona urbana da cidade de Teresina, no Piauí. O estudo faz parte da pesquisa intitulada “Saúde na escola: diagnóstico situacional no ensino médio”^16^. A pesquisa foi desenvolvida por docentes e discentes de um programa de pós-graduação na área de saúde coletiva. Detalhes da metodologia utilizada no estudo-base estão disponíveis na literatura^16^.

Para a seleção dos estudantes, foi utilizada a amostragem probabilística estratificada proporcional^17^, calculada no programa Epi Info 6.04d (*Centers for Disease Control and Prevention*, Atlanta, Estados Unidos), considerando a população de alunos do ensino médio de escolas privadas e públicas estaduais (N = 40.136), segundo dados do Censo Escolar de 2014^18^. Adotou-se intervalo de 95% de confiança (IC95%), prevalência de 50% para os desfechos de interesse para o inquérito, precisão de 5%, efeito de desenho de 1,5 e nível de significância de 5%^19^. Dessa forma, a amostra mínima foi estimada em 571 adolescentes, sendo acrescidos 20% (114) para a possibilidade de perdas e recusas, totalizando a amostra final de 685 adolescentes (Figura 1). Apesar da recusa de 11 alunos (1,6%) em participar da pesquisa, o tamanho amostral não foi comprometido, não havendo perda amostral.

**Figura 1:** Fluxograma da distribuição da amostra de estudantes do ensino médio nas escolas da rede pública estadual e privada de Teresina, Piauí, 2016.

A seleção das escolas considerou o tipo de administração (pública e privada), a localização geográfica (gerências de ensino – Sul, Sudeste, Nordeste e Norte) e o porte (pequeno: até 115 alunos; médio: 116–215 alunos; grande: mais de 215 alunos). Foram selecionadas uma escola pública e uma escola privada de cada porte, distribuídas em cada uma das quatro gerências regionais de ensino da cidade, perfazendo um total de 12 escolas públicas e 12 escolas privadas (Figura 1). A amostra foi distribuída nas escolas sorteadas proporcionalmente ao número de alunos existentes segundo o porte da escola, série do ensino médio, sexo e idade.

A coleta de dados foi realizada no período de abril a setembro de 2016. O horário de coleta dos dados foi determinado pela direção de cada escola, conforme adequação ao calendário e horário de aulas da instituição. O questionário para a coleta de dados foi autoaplicável e composto por seis blocos de perguntas: 1. aspectos sociodemográficos; 2. iniciação sexual; 3. conhecimento objetivo e percebido; 4. aspectos vacinais; 5. aspectos nutricionais e 6. violência e insegurança na escola. Para o estudo em pauta, utilizaram-se questões dos blocos 1 e 6, tendo as perguntas constantes no bloco 6 sido adaptadas a partir do questionário utilizado na Pesquisa Nacional de Saúde do Escolar (PeNSE) de 2012^20^ e do Inquérito de Vitimização utilizado por Lecoque^21^, cuja finalidade foi verificar os fenômenos de violência que se localizam na escola.

Foram considerados vítimas de violência sexual aqueles que responderam sim a pelo menos uma das perguntas a seguir: a) “Nos últimos 12 meses, você se sentiu assediado(a) sexualmente por outros alunos na escola?”; b) “Nos últimos 12 meses, você se sentiu assediado(a) sexualmente por professores ou funcionários na escola?”; c) “Nos últimos 12 meses, você foi forçado(a) ou seduzido(a) a praticar ato sexual (ou foi vítima de violência sexual) por outros alunos na escola?”; d) “Nos últimos 12 meses, você foi forçado(a) ou seduzido(a) a praticar ato sexual (ou foi vítima de violência sexual) por professores ou funcionários na escola?”.

O desfecho do estudo (prevalência da ideação suicida) foi obtido por meio da resposta sim à seguinte pergunta: “Nos últimos 12 meses, você pensou seriamente em cometer suicídio (tirar a própria vida)?”.

Os dados foram submetidos a dupla digitação no programa Epi Info para checar eventuais inconsistências e, quando necessário, realizar as devidas correções. A análise estatística foi realizada no programa IBM *Statistical Package for the Social Sciences* (SPSS) versão 20.0.

Realizou-se análise descritiva, com apresentação da distribuição de frequências relativas e absolutas. A análise inferencial foi realizada para determinar a associação entre a prevalência de ideação suicida e variáveis independentes por meio dos testes do qui-quadrado de Pearson e exato de Fischer (quando aplicável), considerando-se o nível de significância estatística de 5%. Para estimar a razão de prevalência bruta (RP bruta), foi utilizado o método de Mantel-Haenszel para estudos transversais. Para o cálculo da razão de prevalência ajustada (RP ajustada), na qual foram incluídas todas variáveis independentes considerando o nível de significância de p < 0,05, foi utilizado o modelo de regressão de Poisson com variância robusta e IC95%. O poder do teste a posteriori foi calculado no OpenEpi versão 3.01.

O estudo foi aprovado pelo Comitê de Ética em Pesquisa da UFPI (parecer nº 1.495.975). A Secretaria de Estado da Educação e Cultura do Piauí e os gestores das escolas privadas autorizaram a realização da pesquisa nas escolas sob sua responsabilidade. Todos os participantes da pesquisa assinaram o Termo de Consentimento Livre e Esclarecido (TCLE) e o Termo de Assentimento Livre e Esclarecido (TALE), quando necessário.

## RESULTADOS

Participaram do estudo 674 adolescentes (recusa de 11 alunos, 1,6%), cuja média de idade foi de 16,4 anos (DP = 1,2). A amostra foi composta predominantemente por estudantes do sexo feminino (56,7%), negros (77,4%) e que moravam com os pais (85,0%). Com relação à escolaridade da mãe, 68,8% dessas possuíam mais de oito anos de estudo. A maioria dos estudantes declarou não realizar atividade remunerada (83,1%), ter renda familiar maior que um salário mínimo (58,3%) e praticar uma religião (86,8%). Quase sete a cada dez estudantes eram de escolas públicas (64,7%) e a maior parcela cursava o 2º ano do ensino médio (35,9%) (Tabela 1).

**Tabela 1:** Caracterização de alunos das escolas de ensino médio da rede pública e privada de Teresina, Piauí, 2016.

Variáveis	N	%
Total	674	100,0
Idade (em anos)		
14 a 16	346	51,3
17 a 19	328	48,7
Sexo		
Feminino	382	56,7
Masculino	292	43,3
Cor da pele		
Negro^a^	522	77,4
Outros^b^	152	22,5
Mora com os pais?		
Sim	573	85,0
Não	101	15,0
Escolaridade da mãe (em anos de estudo)		
< 8 anos	210	31,2
≥ 8 anos	464	68,8
Atividade remunerada?		
Sim	114	16,9
Não	560	83,1
Renda familiar		
Até 1 salário mínimo	281	41,7
Acima de 1 salário mínimo	393	58,3
Tem religião?		
Sim	585	86,8
Não	89	13,2
Tipo de escola		
Pública	436	64,7
Privada	238	35,3
Ano em que estuda		
1º do EM^c^	222	32,9
2º do EM	242	35,9
3º do EM	210	31,2

EM: ensino médio

^a^ Inclui pretos e pardos

^b^ Inclui brancos, amarelos e indígenas

A prevalência de ideação suicida, nos últimos doze meses anteriores à pesquisa foi de 7,9%, tendo uma maior frequência no sexo feminino (10,2%). Na análise bruta, a ideação suicida esteve associada ao sexo feminino, em frequência duas vezes superior à observada no sexo masculino (RP bruta = 2,13; IC95% 1,18 –3,85; p < 0,05; poder = 66,85%), chegando ao limiar de significância na análise ajustada (RP ajustada = 1,87; IC95% 0,96–3,62; p = 0,052) (Tabela 2).

**Tabela 2:** Prevalência de ideação suicida segundo aspectos sociodemográficos e histórico de violência nos últimos 12 meses entre alunos do ensino médio da rede pública e privada de Teresina, Piauí, 2016.

Variável	Total	Ideação suicida		RP bruta		RP ajustada^a^	
			
	(n = 674)	n	%	IC95%	p-valor^b^	IC95%	p-valor^c^
Ideação suicida	674	53	7,9	-	-	-	-
Idade (em anos)					0,097		0,402
14 a 16	346	33	9,5	1,56 (0,92–2,67)		1,31 (0,70–2,45)	
17 a 19	328	20	6,1	1		1	
Sexo					**< 0,05**		**0,052**
Feminino	382	39	10,2	2,13 (1,18–3,85)		1,87 (0,96–3,62)	
Masculino	292	14	4,8	1		1	
Cor da pele					0,290		0,330
Negros^d^	522	38	7,3	1		1	
Outros^e^	152	15	9,9	0,74 (0,42–1,30)		0,76 (0,43–1,33)	
Mora com os pais?					**< 0,05**		**< 0,05**
Não	101	15	14,9	2,24 (1,28–3,92)		2,27 (1,26–4,10)	
Sim	573	38	6,6	1		1	
Escolaridade da mãe (anos de estudo)					0,278		0,191
≥ 8 anos	464	40	8,6	1,39 (0,76–2,55)		1,55 (0,81–2,97)	
< 8 anos	210	13	6,2	1		1	
Atividade remunerada?					0,453		0,506
Não	560	46	8,2	1,34 (0,62–2,89)		1,28 (0,62–2,66)	
Sim	114	7	6,1	1		1	
Renda familiar					0,793		0,732
Até 1 salário mínimo	281	23	8,2	1,07 (0,64–1,81)		1,10 (0,63–1,91)	
Acima de 1 salário mínimo	393	30	7,6	1		1	
Possui religião?					0,205		
Não	89	10	11,2	1,53 (0,80–2,93)		1,27 (0,64–2,51)	0,495
Sim	585	43	7,4	1		1	
Tipo de escola					0,266		0,279
Pública	436	38	8,7	1,38 (0,77–2,46)		1,38 (0,77–2,50)	
Privada	238	15	6,3	1		1	
Série que estuda							
1º do EM	222	25	11,3	0,71 (0,36–1,39)	0,313	1,86 (0,86–4,03)	0,117
2º do EM	242	17	7,0	1,74 (0,83–3,62)	0,136	1,17 (0,53–2,58)	0,701
3º do EM	210	11	5,2	1		1	
Vítima de violência sexual					**< 0,05**^f^		**< 0,05**
Sim	43	12	27,9	4,30 (2,44–7,55)		3,40 (1,80–6,44)	
Não	631	41	6,5	1		1	
Vítima de violência física					0,123^f^		0,22
Sim	25	4	16,0	2,12 (0,83–5,41)		1,96 (0,67–5,74)	
Não	649	49	7,6	1		1	

RP: razão de prevalência (método de Mantel-Haenszel); IC95%: intervalo de confiança de 95%; EM: ensino médio

^a^ RP ajustada para todas as variáveis independentes (regressão de Poisson).

^c^ Teste do qui-quadrado (95% de confiança).

^b^ Regressão de Poisson com variância robusta (95% de confiança).

^d^ Inclui pretos e pardos.

^e^ Inclui brancos, amarelos e indígenas.

^f^ Teste exato de Fisher (95% de confiança).

A presença de ideação suicida foi associada estatisticamente aos alunos que referiram não residir com os pais (RP ajustada = 2,27; IC95% 1,26–4,10; p < 0,05; poder = 77,23%) e àqueles que informaram ter sofrido violência sexual por outros alunos, professores ou funcionários da escola (RP ajustada = 3,40; IC95% 1,80–6,44; p < 0,05; poder = 99,82%). A frequência de referência à ideação suicida entre estudantes que sofreram algum tipo de violência sexual dentro da escola foi mais de três vezes a de estudantes que não sofreram esse tipo de violência (Tabela 2).

Mesmo não tendo sido verificada associação significativa, é importante ressaltar que as maiores prevalências de ideação suicida foram observadas entre os estudantes mais novos (9,5%), com elevado nível de escolaridade da mãe (8,6%), sem atividade remunerada (8,2%), com renda familiar baixa (8,2%), sem religião (11,2%) e oriundos de escola pública (8,7%). Além disso, ainda que sem significância estatística, houve maior relato de pensamentos sobre o suicídio entre os escolares que sofreram violência física na escola (16%) em comparação àqueles que não foram vítimas dessa violência (7,6%) (Tabela 2).

## DISCUSSÃO

Este artigo apresenta resultados sobre a prevalência de ideação suicida entre adolescentes escolares, possibilitando identificar fatores associados. Percebeu-se que a ideação suicida nos 12 meses anteriores à pesquisa foi de quase 8%, demonstrando-se associada a alguns fatores sociodemográficos e à violência sexual sofrida na escola. Prevalência semelhante foi encontrada em estudo de base populacional realizado com 960 adolescentes na cidade de Pelotas (RS)^11^ e também em Otawa (Canadá)^12^. Ambas as pesquisas mostram características semelhantes ao estudo atual, tendo como público-alvo estudantes adolescentes e desfecho mensurado nos 12 meses anteriores à pesquisa, o que torna este trabalho condizente com a realidade mundial.

Dentre as características sociodemográficas, o sexo feminino e o fato de não residir com os pais apresentaram associação estatisticamente significativa com a ideação suicida. Apesar de a associação com a variável sexo feminino não ter se mantido na análise ajustada, chegando próximo ao limite de significância estatística, a prevalência de ideação suicida foi mais elevada no sexo feminino, o que corrobora evidências já apontadas em outros estudos de que as meninas têm maior propensão à ideação suicida^3,10,12^. Tais estudos têm demonstrado que, apesar de os meninos efetivarem o suicídio em maior proporção, as meninas apresentam maior frequência de pensamentos sobre o ato^3,10,12^.

Um dos fatores relacionados à maior prevalência da ideação suicida entre garotas pode estar ligado ao fato de elas entrarem mais cedo na fase da puberdade, experimentando antes dos garotos as mudanças físicas e pressões próprias do período, especialmente as relacionadas ao que se espera de seu comportamento na sociedade e às repressões familiares que elas sofrem^3,10,12^. O sentimento de solidão e a preocupação com os problemas familiares percebidos no cotidiano de meninas foram relatados em pesquisa realizada por Reis et al.^22^ como uma situação de agravo dos problemas de saúde mental. Ademais, elas apresentam problemas psicológicos mais recorrentes, como a depressão, perturbação de humor, ansiedade e introspecção, fatores que estão fortemente relacionados com o surgimento da ideação suicida^3,23^.

Nesta investigação, a ideação suicida foi mais prevalente entre os adolescentes que não residiam com os pais. A ausência de afeto e apoio familiar revelam um contexto familiar muitas vezes sem comunicação, o que pode gerar sentimentos de abandono e insegurança^24^. Esses sentimentos se tornam a base para o início de um quadro de depressão, que por sua vez é um forte fator para o surgimento de comportamentos suicidas, dentre eles a ideação^23,24^. Os cuidados parentais são a principal base para o bom desenvolvimento social e mental do adolescente^23,24^. Logo, a companhia parental é um laço emocional que deve ser desenvolvido com qualidade desde a infância para garantir o desenvolvimento do adolescente com maturidade psicológica e capaz de enfrentar as várias instabilidades emocionais e/ou psíquicas com mais segurança ao longo da vida^25^.

Outro fator que se destacou no presente estudo foi a maior referência de ideação suicida entre as vítimas de violência sexual na escola. Tal constatação também foi relatada em estudo conduzido no México em 2010^14^, no qual o histórico de abuso sexual na escola aumentou a probabilidade de ideação suicida em 92%. No Brasil, estudo realizado na cidade de Pelotas mostrou que, entre os indivíduos que sofreram violência sexual, o risco de suicídio ao longo da vida esteve presente em 29,2% dos participantes^15^.

A violência sexual, sob a forma de exploração ou abuso sexual, provoca consequências mentais e físicas à vítima, independentemente de sua faixa etária^13^. Pode ser praticada por pessoas que tenham laços afetivos (intrafamiliar) ou pessoas que não possuem parentesco ou relação de convivência com a vítima (extrafamiliar)^13^.

Há evidências de que a violência sexual é mais frequente em vítimas do sexo feminino^15,26,27^. Análise epidemiológica realizada no Brasil sobre a violência sexual mostrou que, no período de 2011 a 2017, 92,4% da violência sexual sofrida por adolescentes notificada no Sistema de Informação de Agravos de Notificação (Sinan) ocorreram no sexo feminino, sendo a residência o local de maior frequência (58,7%), seguido da via pública (14,1%) e da escola (1,2%)^28^. A maior ocorrência da violência sexual no sexo feminino pode estar relacionada a um contexto histórico e sociocultural no qual as mulheres foram criadas e educadas para acreditar que devem ser submissas e aceitar qualquer tipo de dominação^29^.

Qualquer tipo de violência causa impacto na vida das pessoas que as vivenciam, e nos adolescentes afeta o desenvolvimento integral, prejudicando o bem-estar familiar e social^29^. Com relação às consequências da violência sexual, elas podem ser físicas e psicológicas e se manifestar tanto em curto prazo (distúrbios do sono e na alimentação, banhos em excesso, gestos repetidos, quadros ansiosos, isolamento, vergonha, medo e depressão, entre outros) como em longo prazo (abuso de álcool e outras drogas, promiscuidade, disfunções sexuais, baixa autoestima, disfunções menstruais e comportamentos suicidas)^13,25^. A depressão, um dos fatores geradores de comportamentos suicidas entre adolescentes, é uma das manifestações mais recorrentes entre as vítimas de violência sexual, podendo repercutir em qualquer fase da vida, acarretando situações de estresse emocional, social e familiar^15,26^.

A escola é considerada como um espaço de aprendizagem, oportunizando melhores condições de igualdade social e exercendo forte influência na formação dos alunos^30^. As experiências do cotidiano escolar servirão de base para o processo de formação humano de crianças e adolescentes, os quais deverão participar dessas atividades plenamente, a fim de alcançar um desenvolvimento completo^30^. Assim, a escola deve ser compreendida pelos escolares como um local seguro e acolhedor, servindo como meio para o desenvolvimento social, cultural e cognitivo, não devendo, portanto, ser onde os alunos enfrentem qualquer tipo de violência, que possa gerar situações estressoras com impactos que tenham como desfechos as ideações suicidas, uma das consequências mais severas e traumáticas para um indivíduo^3,27,30^.

Sofrer qualquer tipo de violência, inserindo-se aqui a sexual, no ambiente escolar, além das consequências listadas anteriormente, traz prejuízos no aprendizado, pois um aluno vítima de violência, seja ela por professores ou outros alunos, sentirá medo ou vergonha de retornar à escola, impedindo assim o aprendizado de forma integral e desencorajando a pretensão de continuar os estudos^26,30^. Além dos prejuízos educacionais, ser vítima de violência sexual na escola pode provocar prejuízos de cunho social, pois o atraso nos estudos pode gerar sentimentos de baixa autoestima, exclusão e isolamento, bem como prejuízos no âmbito familiar, haja vista que os familiares sofrem a dor e a vergonha juntamente com a vítima^26,27,30^.

Os achados apresentados no estudo evidenciam a importância de se investigar a ideação suicida entre adolescentes escolares, considerando que o desfecho está relacionado a diversos outros problemas e pode gerar consequências maiores, como a morte. Abordou-se um problema atual e crescente, que traz consequências graves para toda a sociedade. Foi relevada forte associação e maior frequência da ideação suicida entre adolescentes do sexo feminino e entre os que não residiam com os pais e destacou-se a elevada referência ao pensamento suicida entre os estudantes que relataram violência sexual dentro da escola.

Os fatores que poderiam enviesar o trabalho foram: a) viés de informação, pois o assunto ideação suicida é cercado de tabus e preconceitos, o que pode ter levado alguns participantes da pesquisa a responder de forma incompleta ou mesmo a deixar de responder a algumas questões, afetando o cálculo da prevalência dos desfechos pesquisados; b) viés de memória, haja vista não ser possível garantir que todos os respondentes lembrassem exatamente de todos os aspectos investigados. Além disso, a ideação suicida é um assunto muito subjetivo e influenciado por múltiplos fatores que não foram analisados neste trabalho, necessitando assim de pesquisas mais detalhadas. Todavia, tais fatores não inviabilizaram a adequada análise do estudo, considerando o desenho metodológico realizado de modo a controlar e reduzir perdas, bem como os achados imprescindíveis para a tomada de decisão na gestão da saúde escolar.

As informações aqui tratadas revelam situações vivenciadas pelos estudantes e que devem despertar a atenção de pais, familiares, profissionais das escolas, gestores do setor saúde e a sociedade em geral para os potenciais riscos advindos da tentativa de suicídio e sua associação com a violência sexual no ambiente escolar. Ambos são problemas de identificação e abordagem complexas, mas que se fazem presentes em nossa sociedade.

É fundamental a divulgação e debate qualificado de informações acerca do assunto e elaboração de estratégias de prevenção que envolvam a família, alunos, professores e outros funcionários das escolas. Devem-se identificar possíveis sinais do comportamento suicida e a possibilidade da ocorrência de violência sexual na escola para que possam ser evitados outros problemas. Frente à importância do tema, recomenda-se a inserção de perguntas relacionadas à ideação suicida e fatores associados nos inquéritos de saúde brasileiros de abrangência nacional, com o intuito de preencher a lacuna de conhecimento existente em relação à realidade dos escolares adolescentes. De posse de um panorama mais abrangente sobre a questão, haverá subsídios suficientes para incrementar as políticas públicas existentes no enfrentamento de um problema complexo e em franco crescimento em todo o mundo.
